# Unveiling Resveratrone: A High-Performance Antioxidant Substance

**DOI:** 10.3390/antiox15010053

**Published:** 2025-12-31

**Authors:** Eunhak Lim, Kyung-Eun Gil, Kyoung-Chan Park

**Affiliations:** 1 Department of Chemistry, College of Natural Sciences, Seoul National University, Seoul 08826, Republic of Korea; kil421@snu.ac.kr; 2 Molecular Innovations Inc., Gwanak-ro 1, Gwanak-gu, Seoul 08826, Republic of Korea; 3 College of Medicine, Seoul National University Bundang Hospital, Seoul National University, Seongnam 13620, Republic of Korea; gcpark@snu.ac.kr

**Keywords:** resveratrone, resveratrol, ascorbic acid, antioxidant, whitening, proliferation, collagen, antibacterial

## Abstract

Resveratrone is a novel compound that was inadvertently discovered by photo-conversion of natural compound resveratrol. Although resveratrol, a representative antioxidant and anti-aging compound, is widely used to promote human health, the benefits of resveratrone have been little studied and remain largely unknown. Since resveratrone has a completely different molecular structure from resveratrol, it has a high possibility of possessing different effects to resveratrol. In this study, the various effects of resveratrone on skin health were revealed, including outstanding antioxidants, whitening, anti-wrinkle, skin regeneration, anti-acne, and so on. Moreover, resveratrone has been confirmed to be an excellent ingredient for skin health because it shows higher performance than resveratrol in most areas.

## 1. Introduction

Resveratrol is a very well-known phytoalexin substance, an antibiotic compound when plants are attacked by fungi or bacteria, which is abundantly found in grapes, berries, buts, and so on [1,2,3]. Based on the previous studies over the past several decades, resveratrol has been recognized as a beneficial substance for health and is thought to be the main cause of the French paradox [4,5,6], because it provides various positive effects on physiological functions, including skin, metabolic, and cardiovascular diseases [6,7,8,9,10,11,12]. Recently, interest in resveratrol for its anti-aging and health benefits has been increasing, as the evidence has emerged suggesting that it can delay aging and promote longevity [13,14].

**Figure 1:**
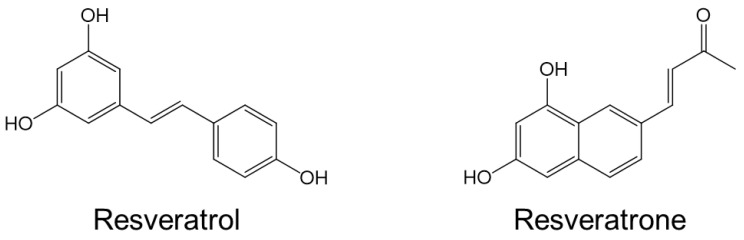
Molecular structure of resveratrol and resveratrone.

As for the molecular properties of resveratrol, it is well known for a long time that *tran*-resveratrol, which is mainly known, is easily transformed into *cis*-resveratrol [15]. However, it has been recently discovered that resveratrol can be photochemically converted into a unique molecule called resveratrone under the special conditions [16]. Since the discovery of resveratrone, it has been shown to be non-toxic, and its spectroscopic properties have been used to enable live imaging of living organisms [17]. However, its dermatological, physiological, biological, and pharmacological effects have been little reported. Because resveratrone has a totally distinct molecular structure from that of resveratrol and does not contain a stilbene structure, it is highly expected to possess significantly different from those of resveratrol (Figure 1).

In this study, the dermatological effects of resveratrone that are primarily associated with improving skin health were investigated. Specifically, it was tested for its effects on antioxidants, whitening, skin regeneration, wrinkle improvement, and anti-acne, which most people are representatively interested in. Because the beneficial effects of resveratrol on the skin have been extensively studied [8,9], all trials were conducted with resveratrol and appropriate control groups included for comparison to demonstrate the superior efficacy of resveratrone. It performed better than resveratrol in almost all experiments, and it even had efficacies that resveratrol did not have. Considering its molecular structure, these findings suggest that resveratrone exerts its physiological and biological effects through a distinct mechanism of action from resveratrol. Also, they indicate that resveratrone exhibits phenotypically superior efficacy compared with resveratrol.

## 2. Materials and Methods

### 2.1. Sample Preparation and Characterization

Resveratrone was synthesized based on the previous report [16]. Resveratrol and ascorbic acid were purchased from TCI (Tokyo Chemical Industry Co., Ltd., Tokyo, Japan) and Sigma-Aldrich, respectively. The synthesized resveratrone was analyzed by GC-FID (5977A MSD, Agilent, Santa Clara, CA, USA) and ^1^H NMR (AVANCE III HD, Bruker, Billerica, MA, USA), showing sufficient purity and ensure its structure (Figures S1 and S2).

### 2.2. DPPH Assay

Approximately 115 μL of 70% ethanol solution was dispensed into each 96-well plate. Approximately 5 μL of each sample was dispensed, and absorbance at 517 nm was measured before the reaction. Approximately 80 μL of DPPH solution was dispensed into each well. Absorbance at 517 nm was measured 10 min after the reaction, and the results were analyzed using pre- and post-reaction absorbance. All results in this work, including DPPH assay, were statically analyzed using the Student’s *t*-test.

### 2.3. Melanin Synthesis Inhibition Assay

B16F10 cells were cultured and an appropriate number of cells was inoculated into each 96-well plate. After 24 h, α-MSH and each sample were treated into all groups except the blank. Each sample was cultured in the culture medium for 72 h. For extracellular assay of melanin synthesis, the absorbance of the cell culture medium was measured. For intracellular assay of melanin synthesis, the absorbance of the supernatant was measured after lysing the cells and separating the supernatant. In both extra- and intracellular assays, the melanin synthesis per cell of each sample was corrected using the BCA method by measuring the each protein content after lysing the cells and separating the supernatant.

### 2.4. Tyrosinase Activity Measurement

An appropriate amount of human-derived normal pigment cells was inoculated into each well and cultured for 1 day. The culture medium of each well was replaced with the culture medium containing each sample and cultured for 72 h. After lysing the cells and separating the supernatant, the absorbance of supernatant at 475 nm was measured at 30 min intervals while reacting with the substrate, DOPA. The measured value of each tyrosinase activity was corrected using BCA method by measuring each protein content after lysing the cells and separating the supernatant.

### 2.5. Cell Proliferation Assay

Human fibroblasts were cultured and an appropriate number of cells were inoculated into well. After 24 h from inoculation, starvation was conducted for 24 h. Cell activity was measured and analyzed using the MTT method.

### 2.6. Collagen Synthesis Assay

Human fibroblasts were cultured and 2 × 10^5^ cells were inoculated into each 6-well plate. Cells were incubated for 1 day in a starvation state. Each sample was treated and cultured for 24 h. After washing, UV light was irradiated and the cells were culture for 48 h. Type Ⅰ collagen expression was measured and analyzed using cell culture medium.

### 2.7. Antibacterial Assay

*Propionibacterium acnes* strains were cultured under anaerobic conditions. The turbidity of the strains was adjusted to McFarland standard No. 1, and the strains were prepared by being diluted 1/20 into liquid culture medium. Each sample was treated into the liquid nutrient medium. Approximately 100 μL of the liquid nutrient medium, which was treated with each sample, was dispensed into each well, and 10 μL of the prepared strains was inoculated into each well. The bacterial counts were measured and analyzed after 72 h of culture under anaerobic conditions.

## 3. Results and Discussion

### 3.1. Antioxidant Activity

**Figure 2:**
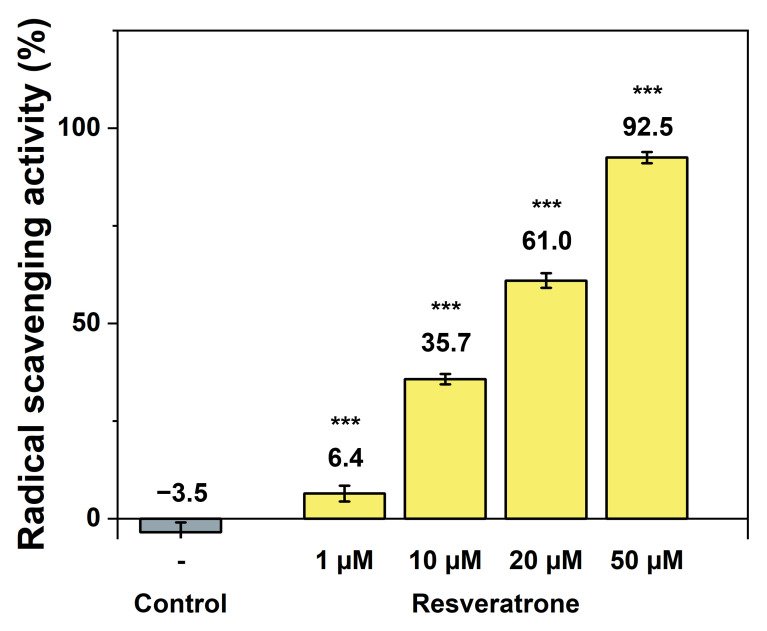
Antioxidant effect of resveratrone measured by radical scavenging activity using DPPH. *** A significant difference at *p* < 0.001 level compared to the control (*n* = 5).

Resveratrol is a well-known polyphenol compound and also a representative antioxidant [12,18,19]. Resveratrone also possesses two phenolic hydroxy groups, suggesting a high potential for its antioxidant properties. Therefore, among various efficacies, its antioxidant activity was investigated first. To measure the scavenging ability of reactive oxygen species and radicals that cause oxidation in the body, the DPPH(2,2-diphenyl-1-picrylhydrazyl) radical scavenging assay was employed. As shown in Figure 2, resveratrone almost completely scavenged radicals at low concentrations, demonstrating its powerful antioxidant properties even at low dose. When compared with resveratrol under the same conditions, resveratrone performed approximately threefold higher radical scavenging activity at the same concentrations (10~20 μM) and showed a significant effect even at low concentrations (1 μM) where resveratrol showed little effect (Figure S3a). Furthermore, since resveratrone demonstrated stronger antioxidant activity than resveratrol, its radical scavenging activity was also compared under the same conditions with that of ascorbic acid (vitamin C), one of the most potent antioxidants. As a result, resveratrone was found to be approximately twice as effective as ascorbic acid at the same concentrations (10~20 μM) and showed a noticeable effect even at very low concentrations (1 μM) where ascorbic acid was ineffective (Figure S3b). In other words, resveratrone exhibited antioxidant effects at lower concentrations than those required for resveratrol or ascorbic acid. At equivalent concentrations, resveratonre showed much stronger antioxidant activity, indicating that it is a highly potent antioxidant.

### 3.2. Inhibition of Melanin Synthesis and Tyrosinase Activity

**Figure 3:**
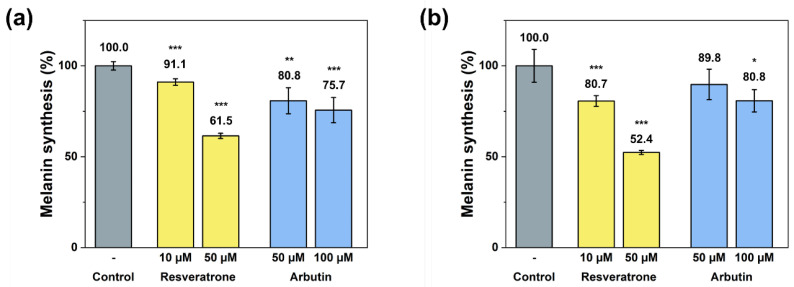
(**a**) Extracellular and (**b**) intracellular inhibition of melanin synthesis by resveratrone and arbutin. */**/*** A significant difference at *p* < 0.05/*p* < 0.01/*p* < 0.001 level compared to the control (*n* = 4).

Since resveratrone has been shown to have excellent antioxidant effects, it is highly likely to affect melanin synthesis, skin whitening, alleviation of pigmentation, and other melanin-related skin symptoms. Melanin, which controls skin color, is synthesized in several steps from the amino acid tyrosine through a catalyzed reaction and radical intermediates are involved in the synthesis of tyrosine into melanin [20]. Hence, resveratrone is highly likely to inhibit the melanin synthesis through its exceptional radical scavenging activity. To confirm the effect of resveratrone on the melanin synthesis, the amount of melanin synthesis in B16F10 cells was measured after stimulated with α-MSH (alpha-melanocyte stimulating hormone). Both extracellular and intracellular measurements of the melanin levels showed that resveratrorne significantly reduced the melanin synthesis (Figure 3). In addition, compared with arbutin, a representative tyrosinase inhibitor [21], resveratrone not only more strongly inhibited melanin synthesis at the same concentration, but 10 μM of resveratrone showed a stronger effect than 50 μM of arbutin in intracellular experiments (Figure 3b).

**Figure 4:**
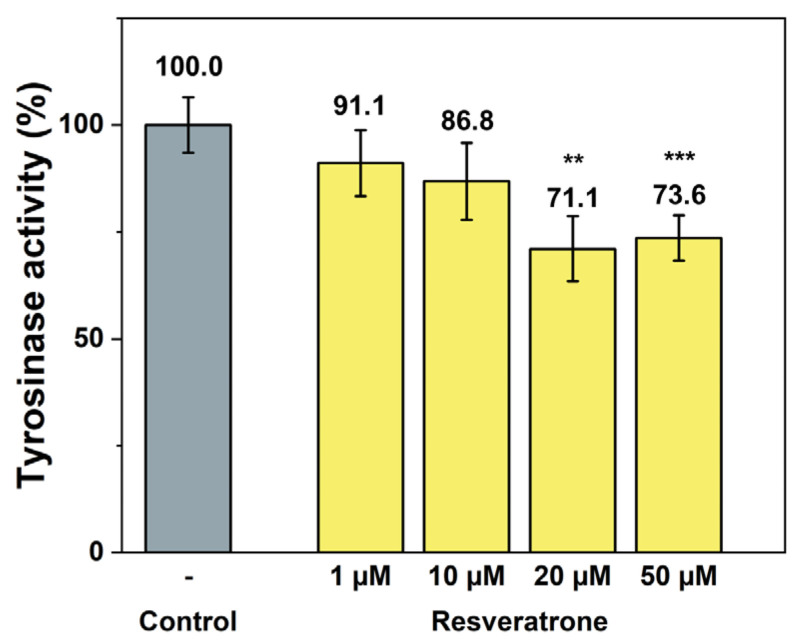
Inhibition of tyrosinase activity by resveratrone. **/*** A significant difference at *p* < 0.01/*p* < 0.001 level compared to the control (*n* = 4).

In addition to the phenotypic inhibition of melanin synthesis, tyrosinase activity assays were also performed to check whether resveratrone specifically interferes with the action of tyrosinase, a key enzyme in melanin synthesis. Resveratrone was found to meaningfully reduce the tyrosinase activity at concentrations similar to those observed in melanin synthesis experiments (Figure 4). Based on the experimental results, it has been demonstrated that resveratrone can suppress the synthesis of melanin by reducing the activity of tyrosinase and has strong antioxidant properties. Although resveratrone showed a relatively lower whitening effect than resveratrol (Figures S4 and S5), it is clearly effective when considering its strong antioxidant power and its superior effect compared with arbutin.

### 3.3. Cell Proliferation and Collagen Synthesis Promotion

**Figure 5:**
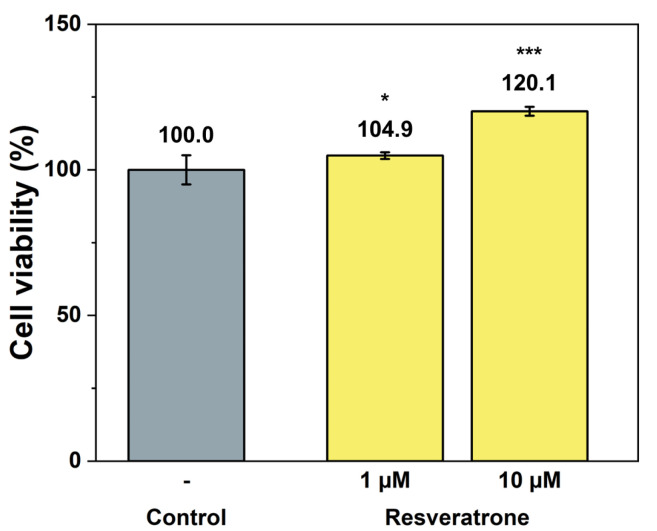
Cell proliferation effect by resveratrone. */*** A significant difference at *p* < 0.05/*p* < 0.001 level compared to the control (*n* = 6).

**Figure 6:**
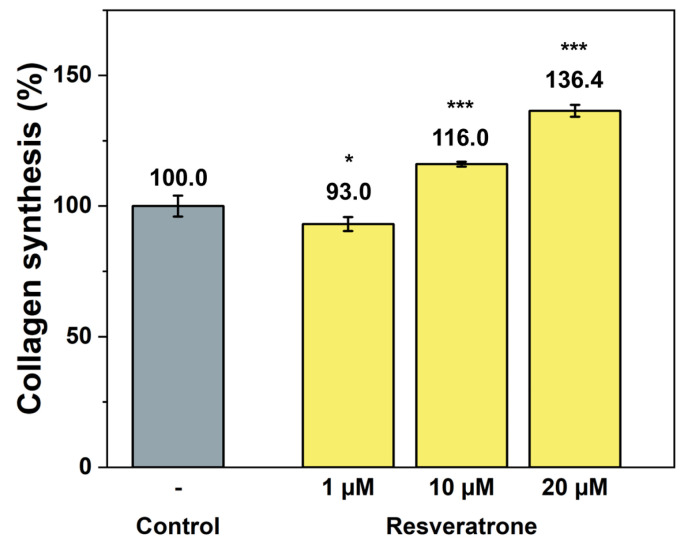
Promotion effect on collagen synthesis by resveratrone. */*** A significant difference at *p* < 0.05/*p* < 0.001 (*n* = 4).

While antioxidants and inhibition of melanin synthesis are closely related to the chemical properties of resveratrone, such as radical scavenging and enzyme reactions, skin regeneration and wrinkle improvement are related to more physiological and biological properties that affect cells and tissues. To examine its effects on wound healing, skin regeneration, skin elasticity, and wrinkle improvement, the activities of resveratrone on cell proliferation and collagen synthesis were tested. The results in Figure 5 and Figure 6 showed that resveratrone not only enhanced the activity of fibroblast but also promoted type I collagen synthesis, suggesting that it increases the activity and metabolism of the entire cell, supporting cellular health. Notably, these effects were not observed in resveratrol. As shown in Figure S6, resveratrone increased cell viability about 20% at 10 μM, but no increase was observed with resveratrol. Similarly, no increase in collagen synthesis was observed with resveratrol up to 20 μM, while resveratrone promoted the collagen synthesis by 36.4% compared to the control group at the same concentration (Figure S7). As expected from the molecular structures of resveratrol and resveratrone, these results clearly support that the underlying mechanism of action of resveratrone is distinct from that of resveratrol, although resveratrone is derived from resveratrol. Moreover, judging by the fact that it shows potentially beneficial effects in humans, which resveratrol lacks, resveratrone may be a more evolved molecule than resveratrol.

### 3.4. Antibacterial Activity

**Figure 7:**
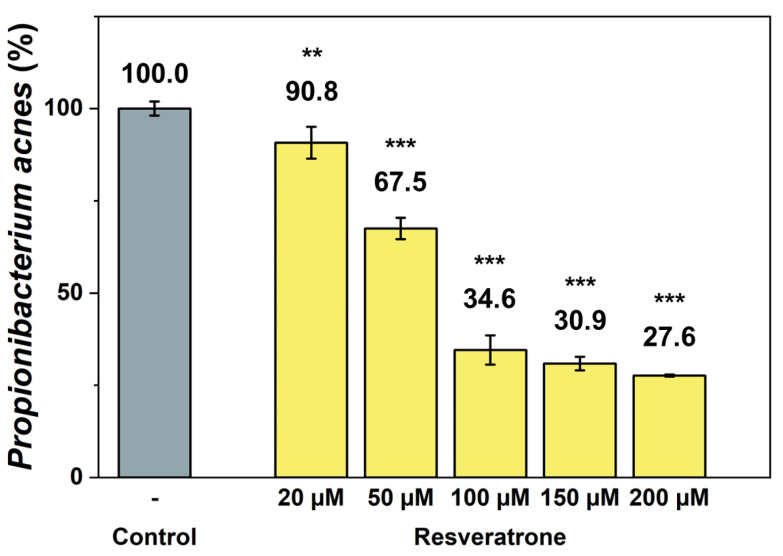
Inhibitory effect of resveratrone on *P. acnes* growth. **/*** A significant difference at *p* < 0.01/*p* < 0.001 level compared to the control (*n* = 4).

Resveratrol not only provides beneficial effects to the body but is also a type of phytoalexin, a natural antibiotic that helps plant protect themselves from foreign substance such as fungi and bacteria [22,23,24,25]. To investigate the effects of resveratrone on external substances related to skin health, its antibacterial activity against the anaerobic acne bacterium *Propionibacterium acnes* (*P. acnes*) was examined, which is most responsible for worsening skin conditions. The results in Figure 7 show that resveratrone effectively inhibits the growth of *P. acnes*. Because the antibacterial effect of resveratrol against *P. acnes* is already well known [25], the efficacy of resveratrol and resveratrone were also compared. Under the same conditions and concentrations, resveratrone was shown to significantly inhibit the growth of *P. acnes* more than resveratrol (Figure S8). Resveratrol did not achieve 50% inhibition even at 200 μM, whereas resveratrone inhibited 65.4% of the bacterial growth at 100 μM. This clearly demonstrates that resveratrone has a superior growth-inhibitory activity against *P. acnes* than resveratrol, suggesting that it not only provides beneficial effects in the body but also has a stronger protective effect against external substances.

## 4. Conclusions

In conclusion, the diverse efficacies of a unique substance, resveratrone, related to the skin health was elucidated through this study. Resveratrone has shown an outstanding antioxidant ability even stronger than resveratrol and even ascorbic acid, so it can remove reactive oxygen species and is expected to have excellent anti-aging properties. In addition to its antioxidant properties, resveratrone was confirmed to inhibit the tyrosinase activity and thereby reduce melanin synthesis, which in turn can contribute to skin whitening and improvement of skin tone. Furthermore, it was confirmed to promote cell activity as well as collagen synthesis, contributing to skin regeneration and wrinkle repair. These effects, which are not observed with resveratrol, suggest that resveratrone provides significantly greater benefits. The finding that resveratrone inhibits *P. acnes* stronger than resveratrol further supports this conclusion. In summary, although resveratrone is derived from resveratrol, it exhibits superior efficacy in most results, making it a highly promising ingredient for improving skin health. Further studies are expected to reveal the clinical effects of the efficacies identified in this study.

## Data Availability

The original contributions presented in this study are included in the article/Supplementary Materials. Further inquiries can be directed to the corresponding author.

## Supplementary Materials

The following supporting information can be downloaded at https://www.mdpi.com/article/10.3390/antiox15010053/s1. Figure S1: GC-FID chromatogram of resveratrone; Figure S2: ^1^H NMR spectrum of resveratrone; Figure S3: Antioxidant effect of (a) resveratrol and (b) ascorbic acid compared to resveratrone under the respectively same condition measured by radical scavenging activity using DPPH; Figure S4: (a) Extracellular and (b) intracellular inhibition of melanin synthesis by resveratrol, resveratrone, and arbutin under same condition; Figure S5: Inhibition of tyrosinase activity by resveratrol and resveratrone under same condition; Figure S6: Cell proliferation effect by resveratrol and resveratrone under same condition; Figure S7: Promotion effect on collagen synthesis by resveratrol and resveratrone under same condition; Figure S8: Inhibitory effect of resveratrol and resveratrone on *P. acnes* growth under the same condition.
