# Supplementary material for: Unveiling Resveratrone: A High-Performance Antioxidant Substance
Source: Antioxidants (Basel). 2025 Dec 31;15(1):53. doi: 10.3390/antiox15010053 (PMC12838096; doi:10.3390/antiox15010053)
Supplement: Supplementary file 1 [file antioxidants-15-00053-s001.zip › antioxidants-4054064-supplementary.pdf]

antioxidants

Supplementary Materials

Unveiling Resveratrone: A High-Performance Antioxidant Substance

Eunhak Lim [1,2,*], Kyung-Eun Gil [1,2] and Kyong-Chan Park [3]

[1] Department of Chemistry, College of Natural Sciences, Seoul National University, Seoul 08826, Republic of Korea
[2] Molecular Innovations Inc., Gwanak-ro 1, Gwanak-gu, Seoul 08826, Republic of Korea
[3] College of Medicine, Seoul National University Bundang Hospital, Seoul National University, Seongnam 13620, Republic of Korea.
* To whom correspondence should be addressed. E-mail: haumir@snu.ac.kr

Supplementary figure

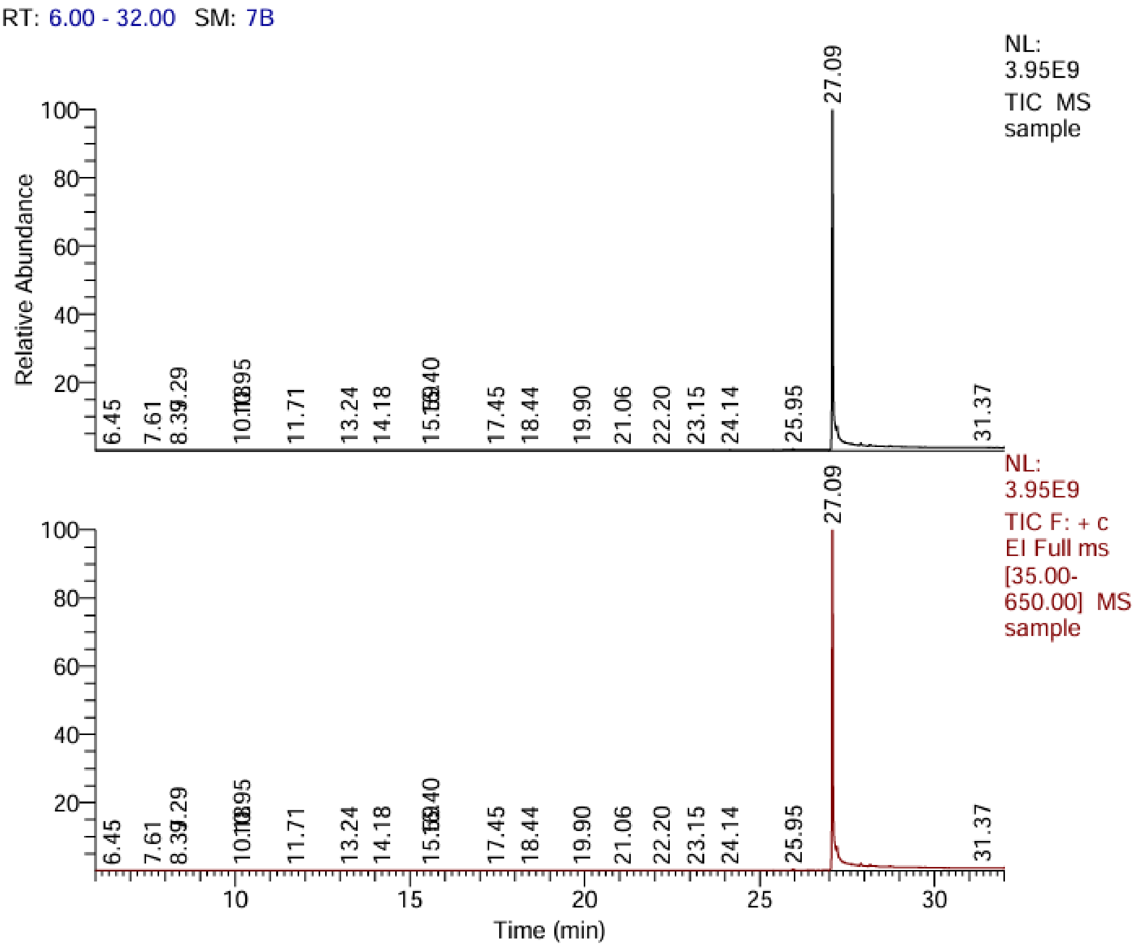

Figure S1. GC-FID chromatogram of resveratrone.

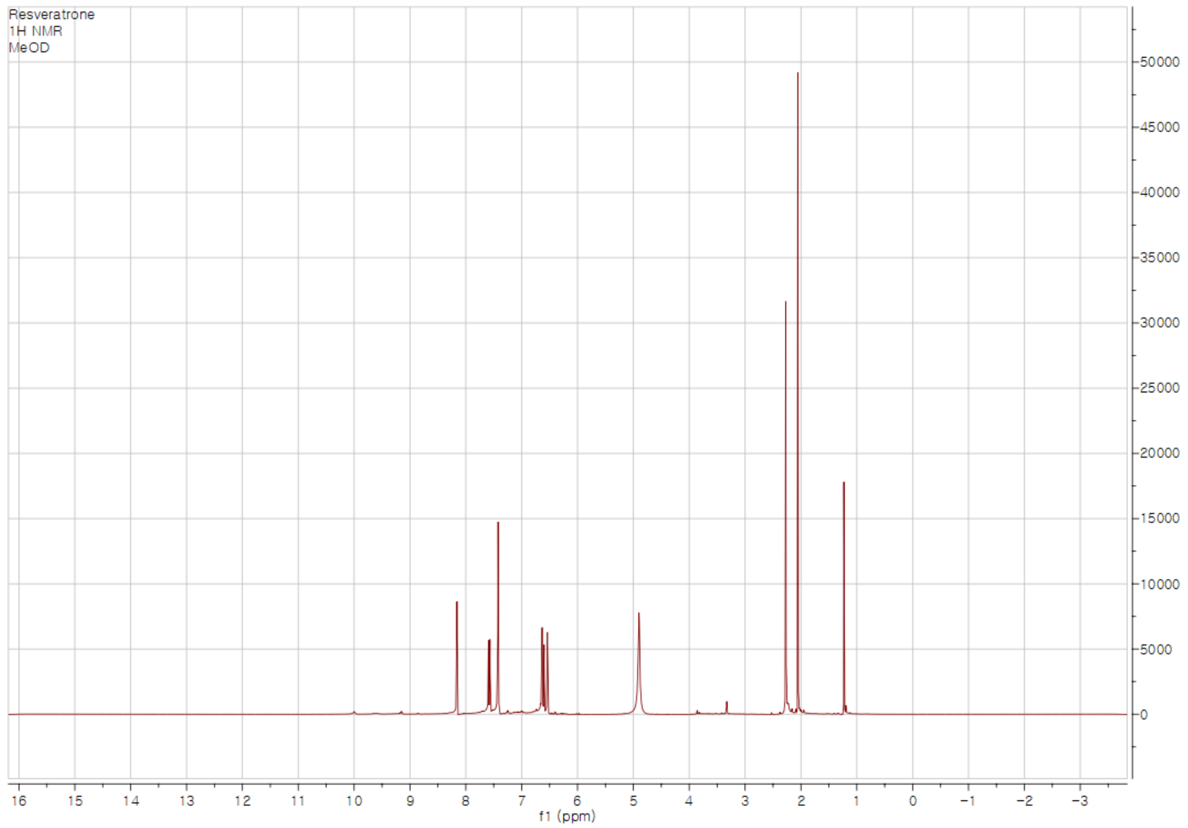

Figure S2. ^{1}H NMR spectrum of resveratrone.

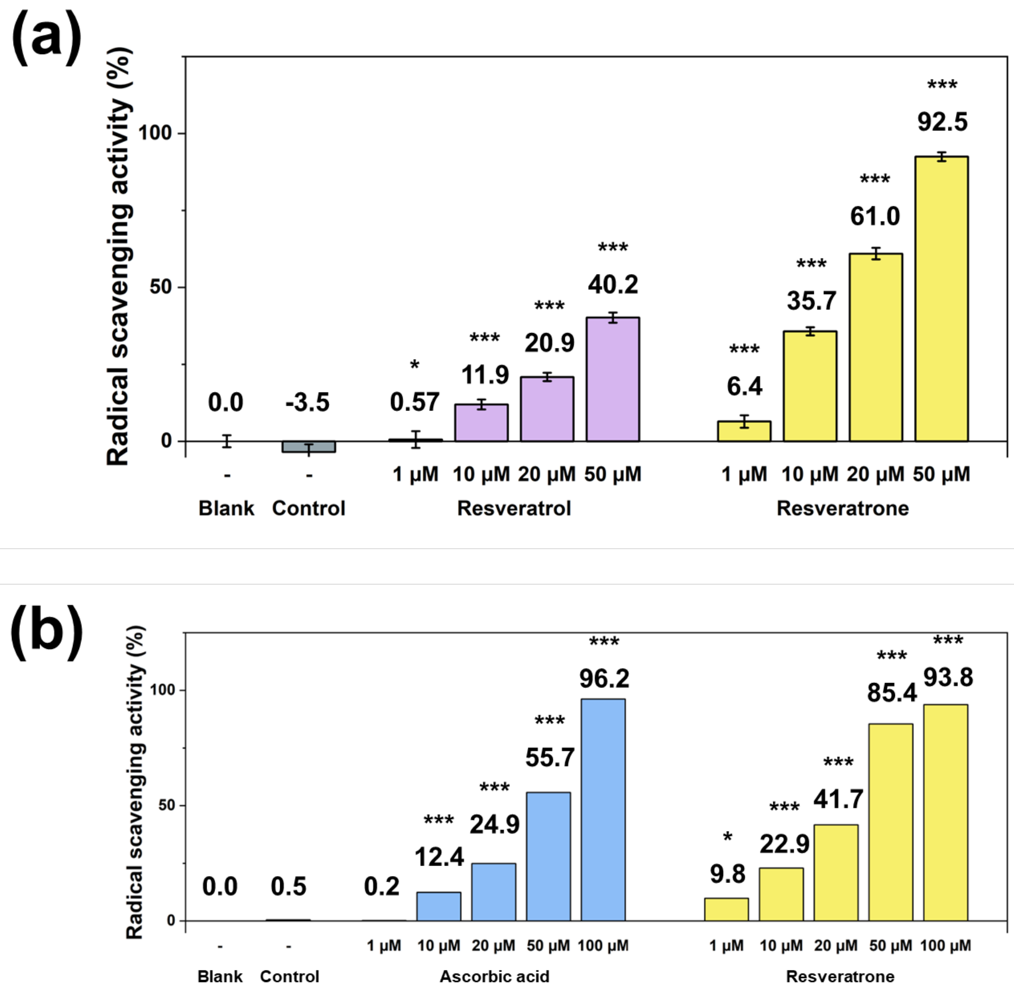

Figure S3. Antioxidant effect of (a) resveratrol and (b) ascorbic acid compared to resveratrone under the respectively same condition measured by radical scavenging activity using DPPH. */*** A significant difference at $p < 0.05/p < 0.001$ level compared to the control ($n = 5$).

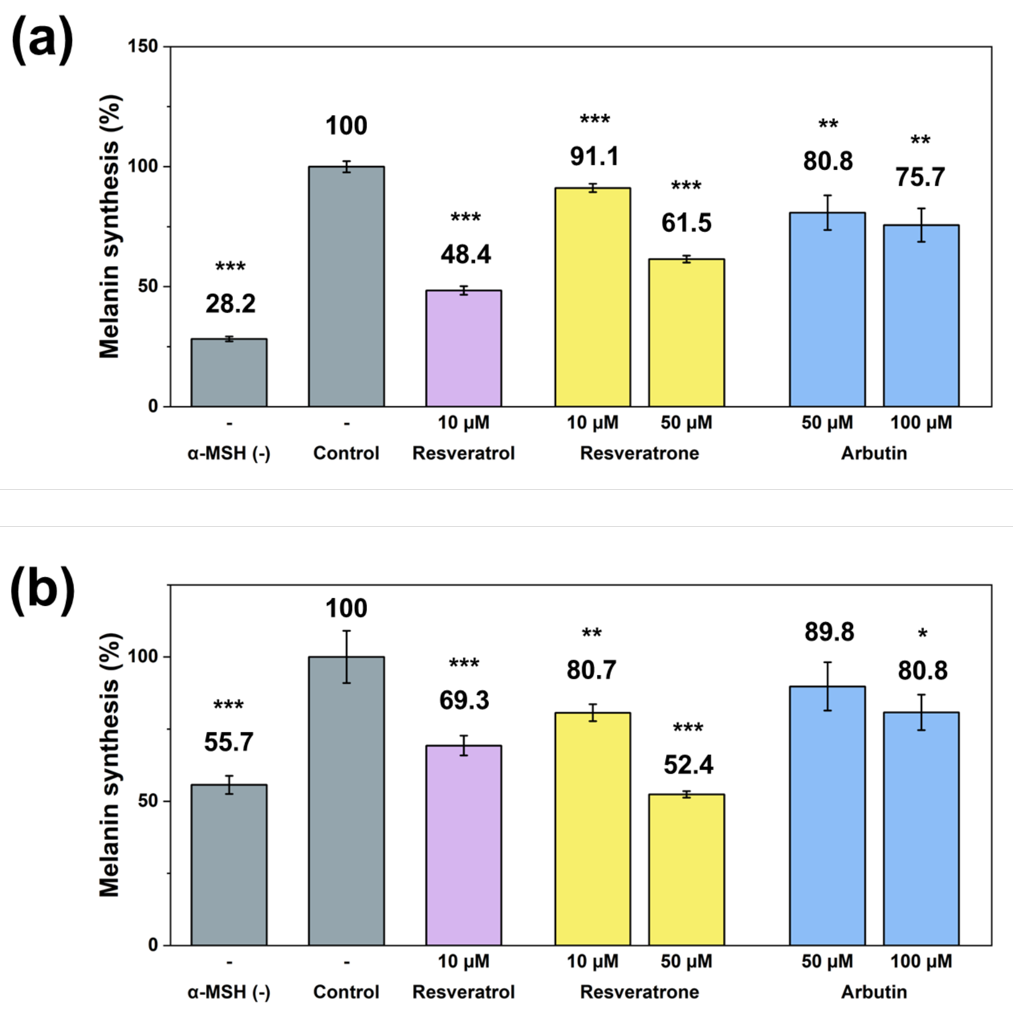

Figure S4. (a) Extracellular and (b) intracellular inhibition of melanin synthesis by resveratrol, resveratrone, and arbutin under same condition. */**/*** A significant difference at $p < 0.05/p < 0.01/p < 0.001$ level compared to the control ($n = 4$).

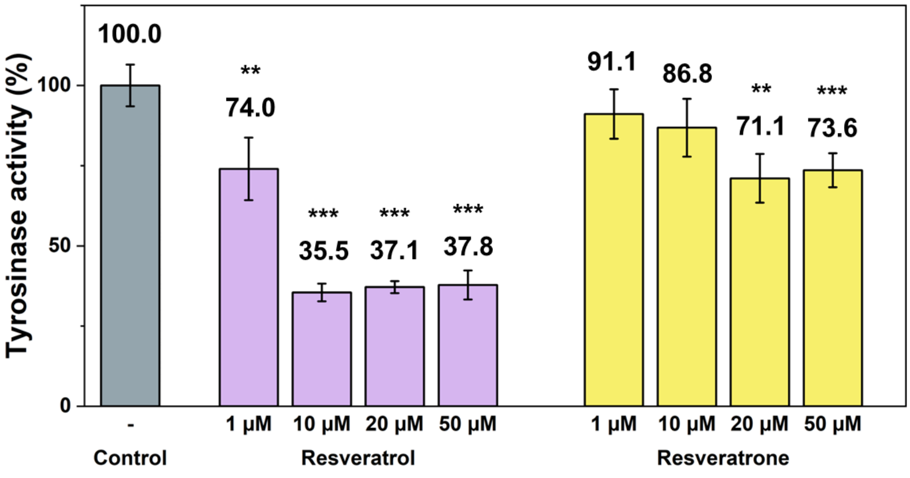

Figure S5. Inhibition of tyrosinase activity by resveratrol and resveratrone under same condition. **/*** A significant difference at $p < 0.01$/$p < 0.001$ level compared to the control ($n = 4$).

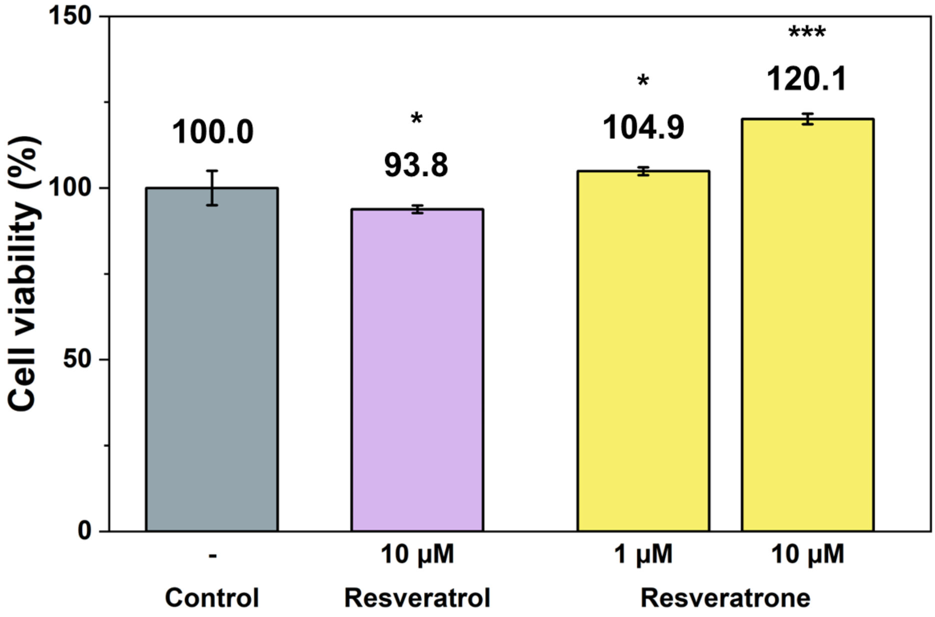

Figure S6. Cell proliferation effect by resveratrol and resveratrone under same condition. */*** A significant difference at $p < 0.05$/$p < 0.001$ level compared to the control ($n = 6$).

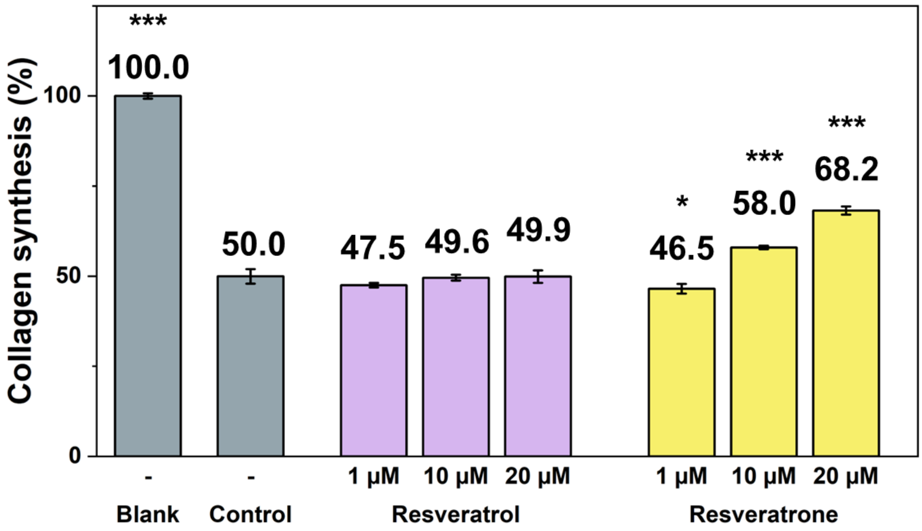

Figure S7. Promotion effect on collagen synthesis by resveratrol and resveratrone under same condition. */*** A significant difference at $p < 0.05$/$p < 0.001$ level compared to the control ($n = 4$).

https://doi.org/10.3390/xxxxx

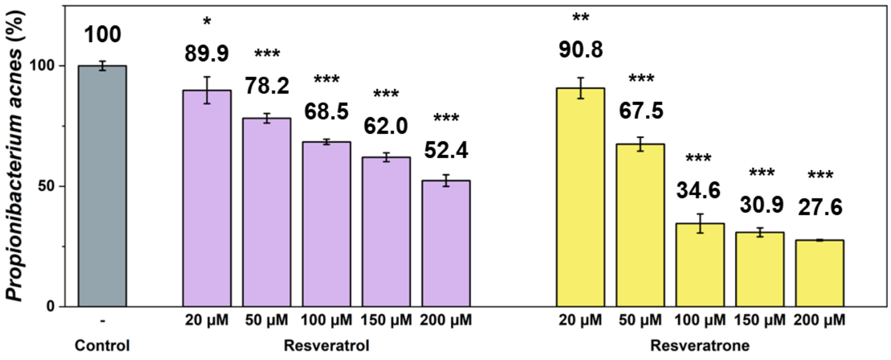

Figure S8. Inhibitory effect of resveratrol and resveratrone on *P. acnes* growth under the same condition. */**/*** A significant difference at $p < 0.05$/$p < 0.01$/$p < 0.001$ level compared to the control ($n = 4$).
